# Decreased mucosal adhesion of *Lactobacillus* species in patients with inflammatory bowel disease

**DOI:** 10.22088/cjim.13.4.71

**Published:** 2022

**Authors:** Saeideh Najafi, Fattah Sotoodehnejadnematalahi, Mohammad Mehdi Amiri, Mohammad Reza Pourshafie, Mahdi Rohani

**Affiliations:** 1 Department of Biology, Science and Research Branch, Islamic Azad University, Tehran, Iran; 2 Department of Immunology, School of Public Health, Tehran University of Medical Sciences, Tehran, Iran; 3 Department of Bacteriology, Pasteur Institute of Iran, Tehran, Iran; # These authors contributed equally to this work

**Keywords:** Attachment protein-encoding genes, inflammatory bowel diseases, * Lactobacillus*

## Abstract

**Background:**

Probiotic *Lactobacillus spp.* modulate immune response via interactions of their binding proteins with epithelial cells. We studied the presence of attachment protein-encoding genes (*mub1*, *mub2,* and *mapA*) in *Lactobacillus* strains with probiotic features isolated from inflammatory bowel disease (IBD) patients and their attachment strength relative to healthy individuals.

**Methods:**

Bacterial strains have been isolated from stool samples of 35 healthy and 23 IBD volunteers. *Lactobacillus spp.* were identified using PCR. Strains with probiotic features were determined by testing resistance against acid and bile. Isolates were assigned as non-adhesive, adhesive, and strongly adhesive strains based on the number of attached bacteria to epithelial cells. Finally, PCR was used to detect the presence of *mub1*, *mub2,* and *mapA* genes.

**Results:**

Probiotic lactobacilli were isolated from 35/35 and 9/23 of healthy and IBD individuals and yielded a total of 87 and 28 strains, respectively. The *Mub1* gene was detected in 95.4% and 100% (p>0.05), *mub2* in 95.4% and 89.3% (p>0.05), and *mapA* in 94.3% and 78.6% (p<0.05) of healthy and IBD isolates, respectively. The numbers of bacteria attached to epithelial cells in healthy and IBD isolates were respectively 33.68±6.00 and 12.23±3.87 in non-adhesive, 71.3±10.83 and 42.17±1.33 in adhesive, 124.40±8.59 and 104.67±5.50 in the strongly adhesive group (p< 0.05).

**Conclusion:**

Less *Lactobacillus* *spp.* with weaker attachments to epithelial cells colonize the gut in IBD than healthy individuals. These findings suggest the beneficial role of probiotics in the management of IBD.

Inflammatory bowel disease (IBD) is a chronic relapsing-remitting inflammatory disorder of the gastrointestinal (GI) tract and often manifests in abdominal pain and diarrhea. IBD refers to two major disorders of Crohn's disease (CD) and ulcerative colitis (UC) with discrete pathologic characteristics (1, 2). Nevertheless, both conditions excessively activate immune cascades on the GI lining. The precise mechanism of immune response overactivation has not been elaborated yet. Firm pieces of evidence have suggested complex interactions between genes, environment, gut microbiota, and the host mucosal immune response (3-5). The prevailing belief is that dysbiosis and the resultant intolerance to commensal intestinal bacteria in genetically susceptible individuals may lead to IBD symptoms (6-8). Mice studies revealed that adhesion of commensal bacteria to the GI mucosa in IBD patients provokes interleukin 17 (IL-17) secretion from T helper 17 (Th17) cells to levels that lead to severe inflammatory response (9-12). Current therapeutic strategies are targeted at symptom control via suppression of immune response (13, 14).

Lately, studies have advocated symbiosis restoration using probiotics as an adjunct treatment to facilitate symptom resolution and as a preventive measure against IBD development (6, 15, 16). Probiotic bacterial species such as *Lactobacillus* are nonpathogenic live microorganisms and regulate immune responses, prevent the colonization of pathogens, and enhance epithelial barrier (17-19). Binding proteins of probiotic *Lactobacillus* species enable them to colonize the GI tract by adhering to the mucosa and epithelial cells and contribute significantly to their favorable activities by virtue of inhibiting the binding of other competitors (20, 21). They further facilitate interactions with the mucosal surface and epithelial cells by subsequently increasing bacterial transition time throughout the gut (22, 23). It is speculated that the adherence of specific bacterial binding proteins to the mucus is also essential to modulate the GI immune system (24, 25).In this regard, numerous binding proteins are spotted in *Lactobacillus* species which are essential to the beneficial actions of probiotics (26). Amid the widely spotted ones, mucus adhesion-promoting protein (MapA) and mucus-binding protein (Mub) have been detected in 86% and 95% of species, respectively (27). 

The Mub proteins are exclusively presented in the probiotic species and consist of repeated functional subunits that are responsible for the adhesive properties of these proteins to GI mucosa (28, 29). MapA is a mucus-stimulating protein that breaks down into antimicrobial peptides and promotes host defense against pathogens (30). MapA was shown to be involved in the binding of *Lactobacillus* species such as *L.* *reuteri* and *L*. *fermentum* to the GI mucus and epithelial cells (22, 23). We hypothesized that the adhesive properties of commensal *Lactobacillus* species isolated from patients with IBD vary from normal individuals. The difference may contribute to the stimulation of exaggerated immune responses in their GI tract. Numerous studies have been conducted on the composition of intestinal microbiota in IBD, but studies on the adhesive properties of these bacteria are scarce. In the current study, we focused on the presence of *mub1*, *mub2*, and *mapA* attachment protein-encoding genes in a mixture of *Lactobacillus* *spp.* isolated from patients with IBD and examined their adhesive properties compared to healthy individuals.

## Methods

**Sample collection: **A total of 550 bacterial strains (given by Pasteur Institute of Iran) that originally had been isolated from the stool samples of 58 individuals with CD, UC, and healthy volunteers were evaluated in this study. The stool samples had been collected from 31 male and 27 female volunteers. The diagnosis of IBD had been confirmed with the combination of clinical presentation, colonoscopy, and biopsy, along with the exclusion of other possible causes of inflammatory GI diseases. In healthy volunteers, stool samples had been obtained from individuals with a normal BMI who otherwise had no history of GI disease, antibiotic use during the previous four weeks, any specific diet, or pregnancy.

Isolates were designated in two arms: IBD arm (obtained from patients with either UC or CD) and control arm (obtained from healthy individuals). Isolates in IBD arm were further divided into two groups:

1. Isolates obtained from new cases of IBD with active disease (AIBD) who had not yet initiated treatments and manifested with diarrhea and/or bleeding and/or abdominal pain, etc.
2. Isolates obtained from IBD patients with controlled disease (CIBD) whose conditions were treated with sulfasalazine or other anti-inflammatory drugs such as corticosteroids and tumor necrosis factor inhibitors and were in clinical remission (no signs and symptoms of active disease).

**Culture condition and screening of isolated bacteria: **All isolates were initially cultured in De Man, Rogosa and Sharpe (MRS) agar medium according to the manufacturer's instructions (Merck Germany). Plates were incubated in an anaerobic condition for 24 hours at 37° C. Following incubation, pure colonies were separated and tested by Gram stain, catalase production, and cell morphology to determine the *Lactobacillus strains *(Gram-positive rods with negative catalase activity).

**Acid and bile tolerance test:** *Lactobacillus spp. *with probiotic features can survive under environmental conditions inside the GI tract and are tolerant of gastric acid and bile. We examined the resistance of strains to acid and bile to isolate ones with probiotic features as previously described (31). To determine the strains resistant to acidic pH, they were grown in MRS broth at 37 °C overnight and centrifuged at 6000 rpm. Pellets were washed in PBS, diluted from 10^-2^ to 10^-10^, platted on MRS agar and incubated at 37° C in anaerobic condition for 48h. Suspensions were counted before pellets were resuspended in 5ml PBS and pH of 3.0 using HCl (Merck, Germany) and incubated at 37°C for 3h. To check resistance to bile, pellets were resuspended in MRS broth containing 0.4% bile salts (Merck, Germany) and incubated at 37°C for 6h. After incubation in bile and acid, the harvested cells were counted. A log reduction of 4 or less was determined as resistant species. 

**DNA extraction: **DNA was extracted from acid and bile resistant *Lactobacillus *strains. The GeneAll DNA extraction kit (Korea) was utilized to perform genome extraction according to the manufacturer's instructions (DNA extraction protocol for gram-positive bacteria). The purity of the extracted DNA was confirmed by a nanodrop spectrophotometer (Thermofisher, USA) at 260 nm and 280 nm.

**PCR to confirm** ***Lactobacillus spp.*** **: **A pair of primers designed by McOrist and colleagues with a nucleotide sequence of F:5’-TGGAAACAGGTGCTAATACCG-3' and R:5’-CCATTGTGGAAGATTCCC-3´ were used to proliferate the *Lactobacillus*-specific gene (32). The PCR reaction mixture with a volume of 25 µL was prepared as follows: 12.5 µL master mix, 1 µL forward primer, 1 µL reverse primer (10 pm / µl concentration), 9.5 µL of distilled water and 1 µL of template DNA. The temperature cycle conditions were applied to Eppendrrof thermocycler (Eppendrrof biotech company, Hamburg, Germany) as follows: an initial heating cycle of 94°C for 5 min, followed by 35 cycles of denaturation at 94°C for 30s, an annealing temperature 55°C for 30s, and an extension cycles at 72°C for 30s, and 72 °C for 7 mins.

**Attachment** **protein-encoding genes** **detection: **The extracted DNAs were used as templates for PCR primers to identify the attachment protein-encoding genes in the *Lactobacillus* strains. The attachment protein-encoding genes studied were *mub1*, *mub2,* and *mapA*. All primers were synthesized by Metabion Co. (Germany). Gene-specific primers used to amplify the attachment protein-encoding genes are shown in table 1. The PCR reaction mixture was prepared for a reaction with a total volume of 25 µL, including 12.5 µL of the master mix, 1 µL of forward primer, 1 µL of reverse primer (10 pm / µl concentration), 9.5 µL of distilled water and 1 µL of template DNA. The temperature cycle conditions were applied to Eppendrrof thermocycler (Eppendrrof biotech company, Hamburg, Germany) as follows: an initial heating cycle of 94°C for 5 min, followed by 35 cycles of denaturation at 94°C for 30s, an annealing temperature specified for each primer according to table 2 for 30s, and an extension cycles at 72°C for 45s, and a final extension cycle at 72 for 10 mins. The final PCR product was then analyzed by gel electrophoresis (1.5% agarose) at 80 V for 40 minutes. The bands were visualized by ethidium bromide staining and photographed after UV treatment by a transilluminator.

**Table 1:** Primer used to detect the presence of Lactobacillus genes involved in binding ability

**Gene**	**Predicted function**	**Primer Sequence (5’- 3’)**	**Melting temperature used (°C)**	**Length**	**Primer references**
*mub1*	mucus-binding protein	F-GTAGTTACTCAGTGACGATCAATG R-TAATTGTAAAGGTATAATCGGAGG	53	150	(44)
*mub2*	mucus-binding protein	F-ACGCGTATTGCGGGTAATGA R-CGCCCCTGAAGTGGGATAGT	55	249	(27)
*mapA*	mucus adhesion-promoting protein	F-TGGATTCTGCTTGAGGTAAG R-GACTAGTAATAACGCGACCG	58	154	(44)

**Cell culture: **HT-29 cells were obtained from the Pasteur Institute of Iran and cultured in RPMI 1640 (Gibco, Germany). HT-29 cells were cultured in RPMI medium containing 10% (v/v) fetal bovine serum (FBS) inactivated by heat (Gibco, Germany), 2% (v/v) L-glutamine 200 mM and 1% (v/v) non-essential amino acids. In addition, 1% (v/v) penicillin and streptomycin antibiotics were added to the medium to prevent contamination. The cells were maintained in flasks (Greiner Bio-One, Strickenhausen, DE) at 37 **°**C and 5% CO2.

**Attachment to GI cells: **Attachment genes are required to be expressed in bacterial cells to exert binding properties to the probiotic bacteria. A more pronounced attachment of bacteria with attachment genes to the epithelial cells relative to the strains which lack these genes, may indicate the possibility of gene expression and the role of related proteins in the observed attachment properties of probiotics. To check the attachment properties of resistant lactobacilli to the GI cells, we examined their ability to attach to the HT-29 cells, which are enterocyte-like cells. HT-29 cells (3 ml of 1.5×10^5^ cells/ml solution) were seeded on 6-well cell culture plates. Once wells became confluent, they were added an aliquot of 2 ml of RPMI (without antibiotics) after washing twice with 3 ml PBS and incubated at 37°C for 3h. Then, bacterial cultures (109 cfu/ml) were added to the wells after suspension in 1ml RPMI1640 medium (without antibiotics). Following the incubation which was performed at 37°C in 5% CO2 for 1h, the wells were washed four times with PBS to remove unattached bacteria. Methanol (1 ml) was used to fixate the attachment. The plates were incubated for 5 – 10 min at room temperature, stained with 3ml of Giemsa stain solution (1:20) (Sigma-Aldrich Co., Mo, USA), and re-incubated for 30 minutes. The number of attached lactobacilli was counted in each well in 20 random microscopic fields. The *Lactobacillus* species in wells with less than 40, 40 – 100 and more than 100 attachments were considered as non-adhesive, adhesive, and strongly adhesive, respectively (33).

**Statistical analysis** **: **The Statistical Program for the Social Sciences (SPSS16, SPSS Inc., Chicago, Illinois, USA) was used for statistical analysis. Continuous variables were expressed as the mean ± standard deviation (SD) or median and were compared using the independent t-test. Categorical variables were expressed as percentages, and differences between groups were judged for significance using the chi-square test. P values < 0.05 were considered significant. The study was approved by the institutional review board (protocol number IR.PII.REC.1398.060) and written informed consent was obtained from all the participants.

## Results

The number of men and women were 19 (54.3%) and 16 (45.7%) in healthy and 12 (52.2%) and 11 (47.8%) in IBD arm, respectively. The difference was non-significant. Among the IBD patients, 18 (78.3%) were diagnosed with UC and 5 (21.7%) with CD. Of 550 bacterial strains, 184 (33%) had phenotypic features of *Lactobacillus* strains (126 were isolated from stool samples of healthy individuals and 58 from IBD patients). Of those, 115 (62%) *Lactobacillus* strains were resistant to acid and bile (determined as probiotic lactobacilli): 87 isolates separated from healthy arm and 28 isolates from IBD arm. Among IBD arm, 25 isolates were from the CIBD group and 3 from the AIBD group. PCR results confirmed that all 115 isolated strains belonged to *Lactobacillus* *spp*. The proportion of stool samples containing lactobacilli with probiotic features and the number of isolated strains from each stool sample in healthy and IBD arm, in AIBD and CIBD groups and UC and CD patients are shown in table 2.

**Table 2:** *Lactobacillus* strains isolated from the healthy arm and IBD arm, CIBD and AIBD arm, and UC and CD patients

	**Healthy**	**IBD**	**P-value**	**CIBD**	**AIBD**	**P-value**	**UC**	**CD**	**P-value**
The proportion of individuals with probiotic *Lactobacilli* strains	35/35 (100%)	9/23 (39.1%)	0.000	7/14 (50%)	2/9 (22.2%)	0.183	7/18 (38.9%)	2/5 (40%)	0.964
Mean number of strains of probiotic *Lactobacilli* isolated from each individual	2.49±1.93	1.22±2.04	0.897	1.79±2.42	0.33±0.70	0.002	1.11±1.77	1.60±3.05	0.235

**Attachment protein-encoding genes detection**: Of the whole isolated acid and bile resistant L*actobacillus* strains, *mub1*, *mub2*, and *mapA* genes were identified in 96.5%, 93.9%, and 90.4%, respectively, by PCR. Proportions of attachment protein-encoding genes detected in isolated *Lactobacillus* *spp.* in healthy, IBD, CIBD, and AIBD samples, and UC and CD patients are shown in table 3. There was no significant difference in the proportions of *mub1* and *mub2* between healthy and IBD arm nor between CIBD and AIBD (p> 0.05).

 However, the proportion of detected *mapA* gene was significantly higher in the healthy arm than the IBD arm as well as in the CIBD group than the AIBD group (p< 0.05). No significant differences in the proportions of detected attachment protein-encoding genes were observed between UC and CD patients.

**Table 3:** Proportions of detected attachment protein-encoding genes in *Lactobacillus* strains

**Genes**	**Healthy**	**IBD**	**P-value**	**CIBD**	**AIBD**	**P-value**	**UC**	**CD**	**P-value**
*mub1*	83/87 (95.4%)	28/28 (100%)	0.248	25/25 (100%)	3/3 (100%)	-	20/20 (100%)	8/8 (100%)	-
*mub2*	83/87 (95.4%)	25/28 (89.3%)	0.239	23/25 (92%)	2/3 (66.7%)	0.180	18/20 (90%)	7/8 (87.5%)	0.847
*mapA*	82/87 (94.3%)	22/28 (78.6%)	0.014	21/25 (84%)	1/3 (33.3%)	0.043	15/20 (75%)	7/8 (87.5%)	0.466

**Figure1:**
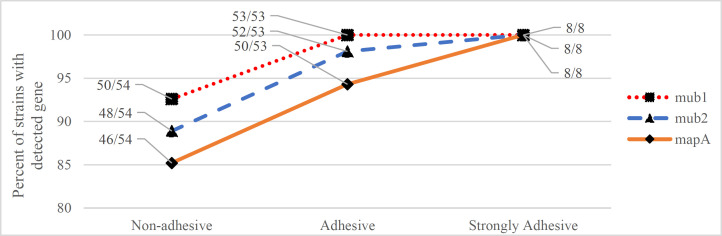
The proportion of detected mub1, mub2, and mapA genes in non-adhesive, adhesive, and strongly

**Attachment to HT-29 cells: **Of the 115 probiotic Lactobacilli isolates, 54 (47%), 53 (46.1%) and 8 (7%) were assigned to non-adhesive, adhesive and strongly adhesive subgroups, respectively; the mean numbers of bacteria adhered to HT-29 cells were 28.52±10.87, 64.74±15.57, and 117.00±8.00 in each subgroup, respectively. Figure 1 depicts the proportion of detected *mub1*, *mub2*, and *mapA* genes in each of these attachment subgroups. An increase in the detection of attachment protein-encoding genes was related to the increasing strength of attachment. All strains with strong adhesion presented *mub1*, *mub2,* and *mapA* genes. A significant increase in the number of bacteria adhered to HT-29 cells was observed in strains in which attachment protein-encoding genes were present in comparison with the ones with absent genes (table 4). 

Moreover, there was a significantly lower number of bacterial attachments in IBD patients than healthy individuals in each attachment subgroup (table 5).

**Table 4:** The number of bacteria adhered to HT-29 cells for each gene

**Genes**	**Not detected**	**Detected**	**P-value**
*mub1*	23.25 ± 9.06	52.38 ± 28.36	0.043
*mub2*	25.29 ± 12.59	53.06 ± 28.34	0.012
*mapA*	24.64 ± 13.07	54.19 ± 28.14	0.001

**Table 5:** The number of bacteria attached to HT-29 cells in healthy and IBD patients at each attachment subgroup

	**Healthy**	**IBD**	**P-value**
Non-adhesive	33.68 ± 6.00	12.23 ±3.87	0.000
Adhesive	71.34±10.83	42.17±1.33	0.000
Strongly adhesive	124.40±8.59	104.67±5.50	0.013

## Discussion

The data presented here emphasizes the role of *mapA*-gene-containing probiotic *Lactobacillus* attachment to the GI lining in the pathogenesis of IBD. We noted that the majority of patients with IBD lose their commensal *Lactobacillus* bacteria with probiotic features. Interestingly, of those who preserved their commensal bacteria, the *mapA* gene was detected in fewer *Lactobacillus* isolates compared to healthy individuals, which indicates a reduced bacterial adhesion potential to the GI mucosa. The reduced potential was confirmed by the smaller number of bacterial attachments to HT-29 cells in IBD isolates. In contrast, *mub* genes were presented equally in almost all strains of IBD and healthy volunteers. Nevertheless, the presence of *mub* and *mapA* genes increased the strength of attachment and the number of bacteria attached to the HT-29 cells in all isolates. Once comparing active and controlled IBD, the number of *Lactobacillus* isolates in controlled patients surpassed those of active patients. Meanwhile, the isolates derived from patients with controlled disease demonstrated the *mapA* gene more than patients with active disease.

The dramatic decrease of isolated *Lactobacillus* *spp*. in IBD patients seen in this study is supported by previous findings in the literature. Particularly, Frank *et. al.,* who comprehensively investigated microbial composition in UC, CD, and healthy individuals through specimens collected from discrete parts of the GI tract, asserted that *Firmicutes* phylum, including *Lactobacillus* species, were depleted in samples obtained from UC and CD patients compared to normal specimens (34). In contrast, Wang *et. al.* reported an increase in *Lactobacillus* in patients with active disease than the healthy group. Moreover, they observed no difference between patients with the controlled disease and healthy ones (35). In our study, apart from that the IBD patients were drained from *lactobacillus* (unlike all healthy individuals who presented *Lactobacillus* in their stool samples), patients with controlled disease had higher quantities of L*actobacillus* than patients with active disease. This suggests the value of some strains of commensal *Lactobacillus* in the management of IBD symptoms. Ganji *et. al.* performed a systematic review of clinical trials on the application of probiotics in IBD treatment. They concluded that a combination of probiotic regimens which include *Lactobacillus* can effectively induce remission in IBD, particularly in UC. In their review, combination regimens demonstrated comparative efficiency for both disease conditions in children (36).

The increased presence of the *mapA* gene in *Lactobacillus* isolates derived from our healthy individuals in comparison with IBD patients, as well as the same observation in patients with controlled disease in comparison with active disease, support the role of *mapA* gene in increasing the effect of probiotic *Lactobacillus* on the regulation of the immune response inside GI tract. It is imaginable that these bacteria gain health benefits from the expression of their *mapA* gene to produce MapA protein. To support this belief, Miyoshi *et. al.* disclosed that intestinal epithelial cells possess a receptor for MapA protein on their surface for the adhesion of *Lactobacillus* (23). 

They observed that blockade of MapA receptor sites on Coco-2 cells (human epithelial colorectal adenocarcinoma cells) inhibits the attachment of *Lactobacillus *to the cells. Enhanced adhesion of *lactobacillus* strains to GI tract mucosa also promotes antagonistic activity against pathogenic bacteria and hinders their growth by competing for adhesion sites (37). For instance, in studies conducted by Turner *et. al.*, they recognized that mapA might be involved in the binding of bacteria to collagen I and fibronectin, which are shared receptors for many pathogens (38, 39). Moreover, Bohle *et. al.* discerned an antibacterial peptide which was a degradation product of MapA protein of L*actobacillus* species obtained from pigs (30). The discovered antibacterial peptide improves the anti-pathogenic properties of species with this protein. 

Over the past decade, the increasing number of data on the molecular origin of adhesion has improved our understanding of the binding properties of *Lactobacillus spp*. In the study conducted by Turbin *et. al.*, *mapA*, *mub1*, and *mub2* genes were detected in 86.5%, 96.5%, and 95.5% of the *Lactobacillus* species, respectively (27). Those numbers were very similar to our findings. Of those, *mub1 *and* 2* genes are exclusive to lactic acid-producing bacteria and present in the highest amounts in *Lactobacillus* species of the GI tract (40). In concordance with our observation, it has been shown that the presence of *mapA* and *mub* genes increases the adhesive strength of *Lactobacillus spp. *to GI epithelial cells (23, 41, 42). Facilitated attachment of bacteria to epithelial cells via MapA may also augment the Mub proteins' interactions with the host. Recently, Xiong *et. al.* disclosed that Mub proteins precipitate aggregation of bacteria and thereby increase their survival. 

These proteins also permit bacteria to modulate the immune response by inhibiting the mitogen-activated protein kinase (MAPK) signaling pathway (20). Studies have reported that Mub proteins downregulate tumor necrosis factor alpha (TNF-α), interleukin-8 (IL-8), IL-1β, IL-6 and IL-12 expression (proinflammatory cytokines) and upregulate IL-10 expression (anti-inflammatory cytokine) (20, 43). No difference was observed between *Lactobacillus* isolates of our healthy and IBD patients in detection of *mub1 *and* 2* genes as all of them presented these genes, which could be due to the importance of these proteins in the survival ability of *Lactobacillus* strains in GI tract.

In conclusion, characteristics of commensal *Lactobacillus* *spp.* in IBD patients differ substantially from those in healthy individuals. The amount of bile and acid-resistant *Lactobacillus *strains and their binding capability decreased in IBD patients' microbiomes. *Mub1*, *mub2*, and *mapA* are binding genes that are shown to increase the adhesive strength of *Lactobacillus spp.* to the intestinal epithelial cells. Among them, the *mapA *gene was less detected in commensal *Lactobacillus* *spp. *of IBD patients. These findings suggest the need for further studies on the possible efficiency of supplementation with certain probiotic *Lactobacillus* *spp.* which possess the *mapA* gene for the prevention and management of IBD.
